# Microstructure and Wear Resistance of In Situ Synthesized Ti(C, N) Ceramic-Reinforced Nickel-Based Coatings by Laser Cladding

**DOI:** 10.3390/ma17153878

**Published:** 2024-08-05

**Authors:** Juncai Li, Ying Chen, Chuang Guan, Chao Zhang, Ji Zhao, Tianbiao Yu

**Affiliations:** School of Mechanical Engineering and Automation, Northeastern University, Shenyang 110819, China; m18524403828@163.com (J.L.); chenyingneu@gmail.com (Y.C.); guanchuang10193@163.com (C.G.);

**Keywords:** laser cladding, titanium alloys, surface modification, nickel-based, ceramic, in situ synthetic

## Abstract

In recent years, laser cladding technology has been widely used in surface modification of titanium alloys. To improve the wear resistance of titanium alloys, ceramic-reinforced nickel-based composite coatings were prepared on a TC4 alloy substrateusing coaxial powder feeding laser cladding technology. Ti (C, N) ceramic was synthesized in situ by laser cladding by adding different contents (10%, 20%, 30%, and 40%) of TiN, pure Ti powder, graphite, and In625 powder. Thisestudy showed that small TiN particles were decomposed and directly formed the Ti (C, N) phase, while large TiN particles were not completely decomposed. The in situ synthetic TiC_x_N_1−x_ phase was formed around the large TiN particles. With the increase in the proportion of powder addition, the wear volume of the coating shows a decreasing trend, and the wear resistance of the surface coating is improving. The friction coefficient of the sample with 40% TiN, pure Ti powder, and graphite powder is 0.829 times that of the substrate. The wear volume is 0.145 times that of the substrate. The reason for this is that with the increase in TiN, Ti, and graphite in the powder, there are more ceramic phases in the cladding layer, and the hard phases such as TiC, Ti(C, N) and Ti2Ni play the role in the structure of the “backbone”, inhibit the damage caused by micro-cutting, and impede the movement of the tearing point of incision, so that the coating has a higher abrasion resistance.

## 1. Introduction

Titanium alloys with excellent performance properties, such as high specific strength, low density, excellent toughness, temperature stability, excellent corrosion resistance, and biocompatibility, have enabled them to perform well and be widely used in the aerospace and automotive industries, chemical engineering, medicine, and metallurgical applications [1,2,3]. However, the limitations, such as low hardness and poor wear resistance, have hindered their wider development and application [4,5]. To further improve the mechanical properties of titanium alloys, many researchers have used surface modification technology to enhance their properties, such as thermal spraying, chemical heat treatment, ion implantation, chemical/physical vapor deposition (CVD/PVD), and laser cladding [6,7]. Among them, laser cladding stands out due to its low heat input and distortion, controlled heat-affected zone, rapid prototyping process, high processing flexibility and repeatability, low material consumption, cost-effectiveness, and strong metallurgical bonding [8].

Titanium carbonitride (Ti(C, N)) is an excellent and versatile non-oxide ceramic. Ti(C, N) is a “zero-dimensional” ternary solid solution containing three phases: TiC_0.2_N_0.8_, TiC_0.3_N_0.7_, TiC_0.7_N_0.3_ [9,10,11]. In addition, TiC has high hardness, while TiN has high toughness. Since TiN and TiC have the same crystal structure (face-centered cubic lattice) and similar lattice constants, they can easily form Ti(C, N) solid solutions. Therefore, Ti(C, N) combines the respective advantages of TiC and TiN, with a series of excellent properties such as a high melting point, high wear and oxidation resistance, high hardness, and corrosion resistance [12].

At present, nickel-based, cobalt-based, and titanium-based alloy powders can be prepared by laser cladding technology to fabricate composite coatings on the surface of titanium alloys. Yu Kun [13] et al. prepared Stellite6-5% TA15-2.5% Y_2_O_3_ and Ni60A-50% Cr_3_C_2_-1.0% Y_2_O_3_ laser fusion coating on the surface of titanium alloy TA15, The former is a cobalt-based coating and the latter is a nickel-based coating. Compared with the substrate, the hardness and wear resistance of both coatings are significantly improved. Laser cladding nickel-based coatings with higher hardness and better wear resistance are increasingly being studied. Chen et al. [14] prepared a composite coating on the surface of titanium alloy TC4with laser cladding technology using Ni, MoS_2_, and Ni60 hybrid powder and explored the effect of the ratio of Ni and MoS_2_ additions on the quality of the coating formation. The experimental results show that the coating has the best performance when the ratio of Ni and MoS_2_ mass fraction is 0.2. The hardness of the coating was increased by 1.8 times and the friction coefficient was reduced by 0.667 times compared with those of the substrate. Zhang et al. [15] prepared WC-Co MMC coatings on the surface of titanium alloy Ti-6Al-4Vwith laser cladding technology, and the deposition material was WC-17Co. The results showed that the coating had good metallurgical bonding with the substrate, the coating was mainly composed of η-Co, W, W_2_C, and WC phases, and the maximum hardness of the coating was five times that of the substrate. Li et al. [16] prepared Ti-Al-Si-Cr-SiC composite coatings on the surface of a TC4 alloy using ultrasonic and rare earth-assisted laser cladding technology. The composite coatings consisted of Ti4Cr, TiC, and Ti_3_Al-Ti_5_Si_3_ eutectic phases, with a refined coating structure, and low dilution. The hardness and abrasion resistance are significantly increased as compared to those of the substrate. Zhao et al. [17] prepared Ti-Zr-B-C laser melting coatings with different B_4_C contents on the TC11 surface. During the laser melting process, TiB, TiC, and ZrC were generated in situ. With the increase in B_4_C content, the number of ceramic reinforcing phases in the coatings increased and were uniformly distributed in the coatings, and the hardness of the coatings increased to 803.8 HV.

Directlyadding ofceramic phases to the coating material or in situ synthesis of ceramic phases during laser cladding are commonly used methods to improve the wear resistance of laser cladding coatings. Naizabekov et al. [18] prepared nickel-based composite coatings with ceramic phases and oxide particles by laser cladding Cu, TiC, and Al_2_O_3_ powders. Hong et al. [19] prepared a nickel-based laser melting coating with ceramic particles by adding TiC particles to Inconel 718 alloy powder, which improved the hardness and wear resistance of the coating. Lv et al. [20] prepared TiC-WC/Ni60 composite coatings with Cr_3_C_2_, Cr_4_Ni_5_W, TiWC_2,_ and other hard phases using laser cladding technology, which effectively improved the hardness and wear resistance of the coatings. Cui et al. [21] synthesized the TiC-TiB_2_ ceramic reinforced phase in situ by adding different contents of B_4_C and nickel cladding powder. As the ratio of C to Ti content increased, the nucleation rate of the ceramic phase in the melt pool decreased and the size of the ceramic phase gradually increased. When the mass fraction of B_4_C was 3% and the mass fraction of graphite was 8%, the coating had the best wear resistance, and the coating wear was reduced by 26.47% compared to the nickel-based coating without additions. Yu et al. [22] added different contents of Ti and B_4_C to the nickel-based powder dr40, and TiC and TiB_2_ ceramic phases were synthesized in situ during laser melting. The in -situ- generated ceramic phases significantly improved the hardness and wear resistance of the coatings. When the mass fractions of Ti and B_4_C were 10%, the hardness of the coatings was increased to 632.66 HV_0.3_, and the wear surface scratches were shallow and uniform. Compared with the mechanical addition of ceramic phases, the ceramic phases synthesized in situ during the laser cladding process are better combined with the coating and more evenly distributed. The laser cladding process of an in -situ- synthesized ceramic phase in nickel-based alloy powder combines the advantages of nickel-based powder and in situ-synthesized ceramic phases, and has a wide range of application prospects and research value.

Titanium nitride (TiN) is a harmless and common metal compound with high hardness and excellent wear resistance. Wang et al. [23] prepared Ni-TiN coatings on the surface of automobile piston pins with the help of laser melting technology. When the power of the laser was 1.5 kW, the ceramic phase was uniformly distributed, the coating structure was dense, and the microhardness reached 843.2 HV, with good wear resistance. Liang et al. [24] prepared an AlCrNiTi(Fe, Cu) laser fusion coating containing a TiN reinforcing phase. TiN ceramic particles were synthesized in situ and randomly distributed in the composite coating, which served to refine the grains and increase the lattice constant. Compared with the AlCrNiTi(Fe, Cu) coating synthesized without TiN, the hardness of this coating was increased by 33.33%, the wear was reduced by about 50%, and the generation of TiN ceramic particles significantly improved the mechanical properties of the coating. You et al. [25] prepared a Ti-Al-N composite coating containing the Ti_2_AlN phase on the surface of a titanium alloy using the laser cladding technique. The coating contained TiN, Ti_2_AlN, and other hard ceramic phases, and the hardness of the coating was about doubled compared to the substrate. Feng et al. [26] prepared Ti-Al-(C, N) coatings by laser cladding technology; the enhanced phases of the coatings were fine and uniformly distributed, and the hardness of the coatings was significantly improved compared to the substrate. This process is expected to improve the surface mechanical properties of the workpiece to adapt to the complex working conditions during the service of the workpiece.

The above studies show that although the simple addition of single ceramic particles can optimize the organization and properties of laser-clad nickel-based alloy cladding layers to a certain extent, there are still limitations. For example, due to the differences in density, compatibility, lubricity, thermal expansion coefficient, and modulus of elasticity between the ceramic particles and the cladding material [27], these factors may lead to the deposition of ceramic particles in the cladding layer as well as the generation of stress, which may in turn affect the performance and reliability of the cladding layer. In practical applications, it is necessary to select the appropriate coating material and preparation process according to the specific needs and environmental conditions [28].

Many scholars have studied composite coating with single ceramic phases, such as TiC, WC, SiC, and TiN, but the study of laser cladding in situ synthesized Ti(C, N) ceramics Ni-based composite coating on titanium alloy is still rare. In addition, most of the research in academia and the industry focuses on laser melting of pre-positioned mixed powders, and due to the poor fluidity of powder mixtures, there are even fewer studies on laser melting coaxial powder feeding for the preparation of in situ synthesized Ti(C, N) ceramic phase coatings. Therefore, in this study, to improve the wear resistance of titanium alloy materials, we chooses In625 powder, TiN, graphite, and pure Ti powder, and prepared nickel-based composite coatings with in situ synthesized Ti(C, N) ceramic phase by coaxial feeding laser melting. The feasibility of possible chemical reactions was determined by thermodynamic calculations, and the elemental composition and physical phases of the fused coatings were analyzed by SEM, EDS, and XRD. Finally, the wear resistance of the prepared coatings was measured by a multifunctional friction and wear tester and 3D laser confocal microscopy. The present study was carried out to improve the hardness and wear resistance of titanium alloys and to provide theoretical and technological support for their further applications.

## 2. Methods and Materials

### 2.1. Preparation and Properties of Material Samples

In625 alloy powder was chosen as the initial powder. Meanwhile, to improve the performance of the titanium alloy substrate, four composite powders with different weight ratios were designed to gradually increase the content of ceramic particles synthesized in situ in the composite coating, as shown in Table 1. The labels 1#–4# in Table 1 denote samples 1–4, respectively. A high-precision digital electronic balance (0.01 mg) was used to weigh each powder, and the compositions of the composite powders used to prepare the samples as well as the process parameters are shown in Table 1. To mix the respective powders well, the mixed powders were placed in a ball mill for two hours at 450 r/min, wherein the mass ratio of the alumina ball to the composite powder was 2:1. Subsequently, the impurities in the composite powder were removed using an 80 mesh. Before the experiment, the composite powder was dried in a constant-temperature oven at 80 °C for 2 h to remove the moisture, to increase the fluidity of the powder, to avoid vaporization, and to reduce the porosity defects. The titanium alloy with a length, width, and height of 10 × 5 × 4 cm^3^ was chosen as the experimental substrate. Its chemical composition is shown in Table 2.

### 2.2. Experimental Setup for Laser Cladding

**Figure 1:**
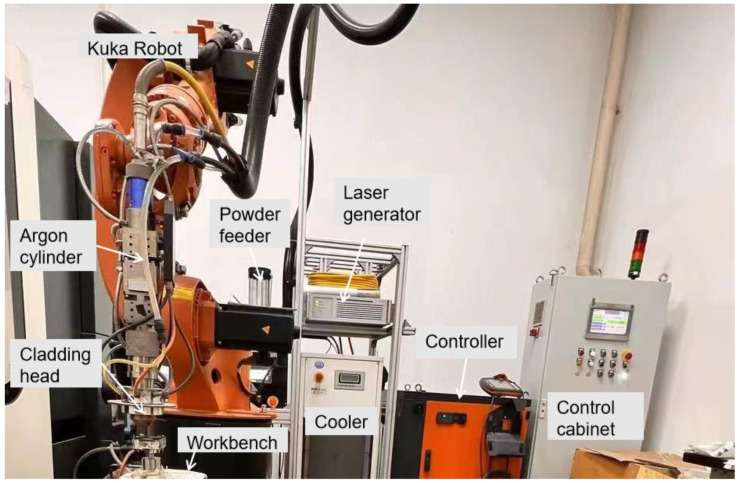
Experimental setup diagram of laser cladding system.

The equipment for this study was a coaxial LEDED system, which mainly consists of a laser generator, powder feeder, chiller, KUKA robot, laser cladding head, etc., as shown in Figure 1. The maximum power and wavelength of the CW fiber laser are 500 W and 1070 nm. Before the laser cladding, the surface of the substrate was polished and then cleaned with alcohol to prevent impurities and rust on the surface. The distance from the top of the substrate to the bottom of the powder feeding nozzle was 15 mm to ensure the coincidence of the deposition plane and the laser focal plane and to improve the laser energy and powder capture efficiency. The diameter of the laser beam on the deposition plane was about 1.0 mm. Inert gas of Ar with a purity of 99.99%, was used for both the protection gas and the powder feeding gas, with flow rates of 20 L/min and 8.0 L/min, respectively.

### 2.3. Tribological Tests and Tribometers

**Figure 2:**
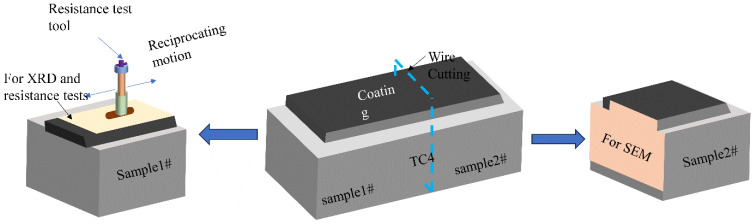
Preparation process of test samples.

Figure 2 shows the preparation of the test samples. At the end of the experiment, each sample was cooled to room temperature (−20 °C) in air. Sample block 1# and sample block 2# were cut from the substrate using the EDM technique and used for mechanical property testing and microstructure observation of the composite coatings, respectively. Planes 1 and 2 were polished with SiC sandpaper with grit sizes ranging from 400 mesh to 3000 mesh. Subsequently, the planes were polished to a mirror-like surface with diamond polishing paste with a grit size of 2 μm, and then washed and blown dry. Plane 2 was sanded and polished and then placed in HF acid etching solution (HF:HNO_3_:H_2_O = 1:1:10) for 10–15 s. Before testing, all sample blocks were placed in alcohol and ultrasonically cleaned for 2 min to remove surface dirt, and finally, the samples were blown dry with an air blower.

The phase composition was analyzed by X-ray diffractometry (XRD, X-Pertpro, The Netherlands, Cu-Kα target) on plane 1 with a 2θ range of 20° to 90° and a scan time of 10 min. The size, color, and distribution of phases of the composite coating were examined by SEM and EDS on plane 2. A multifunctional material surface property tester (MFT-4000) was used to conduct friction and wear tests on plane 1 to analyze its wear performance. Reciprocating friction tests were performed on plane 1 at room temperature using Al_2_O_3_ balls with a diameter of 5 mm (hardness > 90 HRC). The specific parameters were a normal load of 15 N, a unidirectional stroke length of 5.0 mm, a speed of 200 mm/min, and a time of 50 min. The entire wear trajectory was observed with OLYMPUS laser confocal microscopy (OLS4100, Olympus Corporation, Tokyo, Japan) and used to calculate the wear volume.

## 3. Results and Discussion

### 3.1. Thermodynamic Calculations

Due to the addition of TiN, graphite powder, and pure titanium powder to the initial composite powder, In625, the main elements in the molten pool are Ni, Ti, Nb, C, N Cr, etc. The possible chemical reactions during the solidification process of the laser cladding are as follows:

1# Ti+C→TiC

2# Nb+C→NbC

3# $$7\mathrm{Cr}+3C\to {\mathrm{Cr}}_{7}C_{3}$$

4# $$3\mathrm{Cr}+2C\to {\mathrm{Cr}}_{3}C_{2}$$

5# $$\mathrm{Ti}+3\mathrm{Ni}\to {\mathrm{TiNi}}_{3}$$

6# Ti+Ni→TiNi

7# Ti+N→TiN

8# $$x\mathrm{Ti}C+(1-x)\mathrm{Ti}N\to \mathrm{Ti}=\mathrm{Ti}(C_{x},N_{1-x})(0<x<1)$$

9# $$\mathrm{TiN}+C\to \mathrm{TiC}+\frac{1}{2}N_{2}$$

The Gibbs free energies ($\Delta G_{T}^{\Theta }$) of reactions 1#–9# were calculated as follows. The Gibbs free energies of the reactions were used to determine the direction in which the chemical reactions proceeded in the in situ synthesis, and the $\Delta G_{T}^{\Theta }$ of each reaction could be calculated from the enthalpies of the reactants or products at the temperature of *T* and the standard entropy difference of the reaction. The $\Delta G_{T}^{\Theta }$ is calculated as follows [29,30]:

(1) $$\Delta G_{T}^{\Theta }=\sum G_{\mathrm{product}}^{\Theta }\left(T\right)-\sum G_{\mathrm{reactant}}^{\Theta }\left(T\right)$$

**Figure 3:**
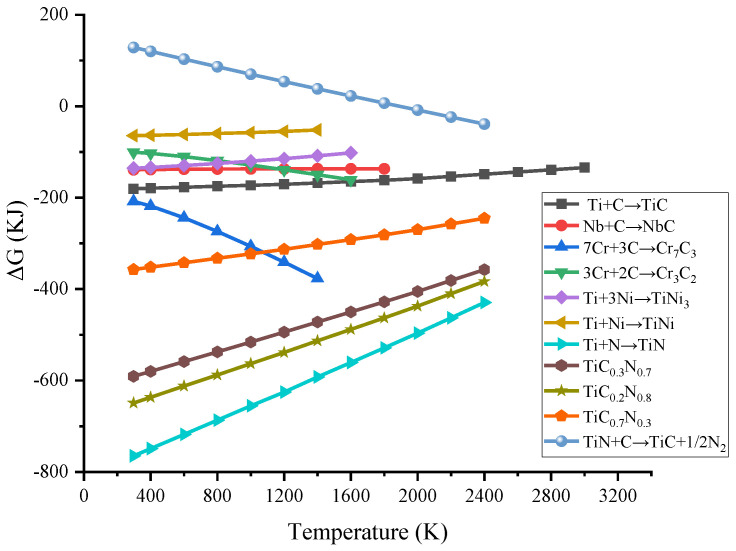
Temperature—dependent Gibbs free energy curves for various potential reactions during the solidification process.

The right two terms of Equation (1) are the $\Delta G_{T}^{\Theta }$ of the products and reactants, respectively, and can be expressed as

(2) $$G_{i}^{\Theta }(T)=H_{i}^{\Theta }-T\cdot S_{i}^{\Theta }(T)G_{i}^{\Theta }(T)$$

where $H_{i}^{\Theta }(T)$ denotes the enthalpy of the reactants or products at a temperature of *T*. $S_{i}^{\Theta }(T)$ denotes the standard entropy, which can be calculated using Equation (3):

(3) $$S_{i}^{\Theta }(T)=\Delta_{f}H_{i}^{0}(298)+\int_{298}^{T}C_{\mathrm{Pi}}(T)dT+\Delta H_{i}^{\Theta }(T_{m})$$

where $\Delta_{f}H_{i}^{0}(298)$ denotes the entropy of substance *i* at the temperature of 298 K, and $\Delta H_{i}^{\Theta }(T_{m})$ denotes the latent heat due to the phase transition of substance *i*. Since the crystal structures of TiC and TiN are very similar, it is very easy to form a Ti(C, N) solid solution, and its Gibbs free energy can be calculated by the following equation [30]:

(4) $$\Delta G_{T}^{\theta }[{\mathrm{TiC}}_{x}N_{1-x}]=x\Delta G_{T}^{\theta }[\mathrm{TiC}]+(1-x)\Delta G_{T}^{\theta }[\mathrm{TiN}]+RT\left\{x\ln^{x}+(1-x)\ln^{\left(1-x\right)}\right\}$$

where *R* is a universal constant taking the value 8.314 K·J^−1^, and *x* is commonly 0.3, 0.2, or 0.7. The material parameters used in the thermodynamic calculations were taken from the thermodynamic handbook of the Handbook of Inorganic Materials. According to the thermodynamic criterion, the direction as well as the order of chemical reactions can be judged by the magnitude $\Delta G_{T}^{\Theta }$ of each reaction. The curve of $\Delta G_{T}^{\Theta }$ versus temperature (less than the corresponding melting point) for each potential reaction from 1# to 9# was obtained by calculation, as shown in Figure 3.

From the $\Delta G_{T}^{\Theta }-T$ curves, it can be found that the $\Delta G_{T}^{\Theta }$ of reaction (9#) is more than when the temperature is in the range of 298–1890 K, which indicates that reaction (9#) is not favored. However, when the temperature is above 1890 K, the Gibbs free energy indicates a positive progression of reaction (9#), which implies that graphite can replace [N] in TiN and form TiC in the outer layer of TiN. Except for reaction (9#), the $\Delta G_{T}^{\Theta }$ of all potential reactions is less than 0 when the temperature is greater than 0 and lower than the respective melting points, indicating that these potential reactions can proceed spontaneously. Based on the magnitude of the $\Delta G_{T}^{\Theta }$, the priority of the reactions during solidification is 7# > 8# > 3# > 1# > 2# > 4# > 5# > 6# in order of magnitude without considering the concentration of each substance. Therefore, it is feasible to generate Ti(C, N)-reinforced nickel-based composite coatings under the given thermal reaction conditions utilizing this powder ratio. During the in situ formation of Ti(C_x_, N_1−x_), the $\Delta G_{T}^{\Theta }$ versus temperature curves of the three phases are very similar and all are much smaller than 0. As *x* decreases, $\Delta G_{T}^{\Theta }$ gradually increases, and thus Ti(C_0.2_, N_0.8_) is relatively easy to form among the three phases.

### 3.2. XRD and EDS Analysis

**Figure 4:**
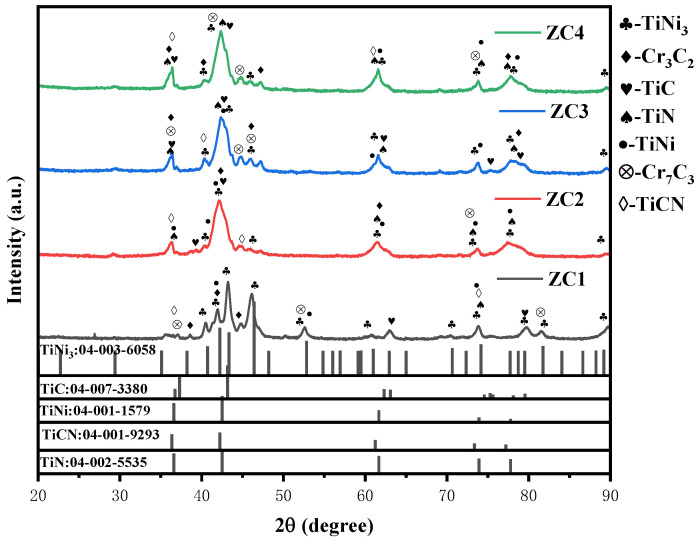
X-ray diffraction (XRD) patterns of different samples.

The X-ray diffraction (XRD) patterns of the different samples are shown in Figure 4. The prepared coatings were mainly composed of TiC, TiN, Ti(C, N), and Ti-Ni phases. The addition of TiN ceramic, pure titanium powder, and graphite to samples 1# and 2# produced TiC phases with diffraction peaks at 37.306°, 43.177°, and 63.103° [PDF#04-007-3380(RDB)], which is consistent with standard XRD cards. With the increase in TiN and graphite additions, the diffraction peaks of Ti(C, N) increased, and the diffraction peaks at 36.353°, 42.225°, 61.248°, 73.358°, and 77.202°are consistent with the standard XRD cards of [PDF#04-001-9293 (RDB)]. From the previous thermodynamic calculations, it can be seen that C can displace [N] in TiN at 1900K, forming Ti(C, N) in the outer layer of large-grained TiN. The crystal structures of TiC and TiN are very similar, and small-grained TiN can form Ti(C, N) solid solution directly.

**Figure 5:**
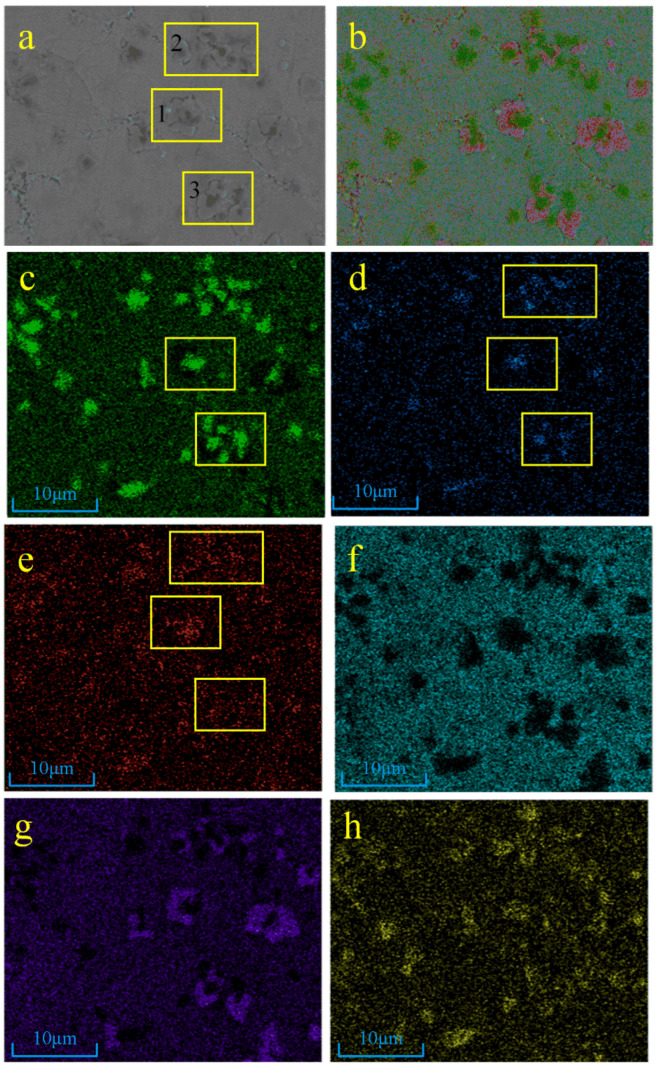
EDS results of sample 1#: (**a**) electronic image, (**b**) EDS layered image, (**c**) Ti element, (**d**) N element, (**e**) C element, (**f**) Ni element, (**g**) Cr element, (**h**) Nb element.

The EDS surface scan of sample 1 is shown in Figure 5, and it can be found by the distribution of Ti, N, C, Ni, and Cr elements that the microstructure of sample 1# mostly consists of Ti/Ni and C/Cr generators. Due to the relatively low additions of TiN, Ti, and graphite in the powder, there are no large TiN particles in the microstructure. From Figure 5c–e, it can be seen that there is a high degree of overlap of Ti, N, and C in regions 1–3 (yellow boxes 1–3 in Figure 5), which is judged to be the melting of small TiN particles to form a Ti(C, N) solid solution.

**Figure 6:**
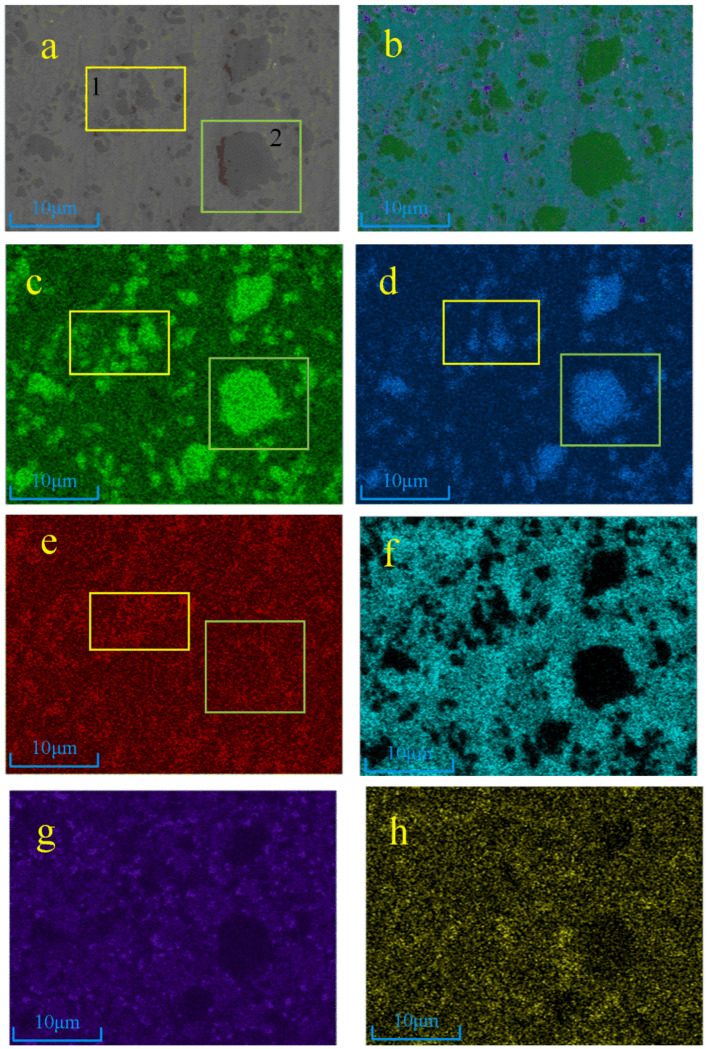
EDS surface scan image of sample 4#: (**a**) electronic image, (**b**) EDS layered image, (**c**) Ti element, (**d**) N element, (**e**) C element, (**f**) Ni element, (**g**) Cr element, (**h**) Nb element.

The EDS results and the distribution of Ti, N, C, Ni, and Cr elements in sample 4 are shown in Figure 6. It can be seen that due to the high addition of TiN, Ti, and graphite in the powder, the distribution of Ti and N elements overlap well. It can be judged that the black dots and lumps in the electron diagram are TiN particles, region 1 (box 1 in Figure 6) is small TiN particles, and region 2 (box 2 in Figure 6) is large TiN particles. Element C is densely distributed around the small black lumps and large black masses. From Figure 6c–e, it can be seen that the three elements of Ti, N, and C are enriched in region 1. Combined with the previous XRD and thermodynamic analyses, it can be judged to be a Ti(C, N) solid solution formed with C elements after the melting of small-grained TiN. C is present at the periphery of the large particles of TiN in region 2, while a small area in the lower right corner is observed to contain Ti, C, and N. Combined with the XRD results, it can be determined to be a Ti (C, N) phase, and it is more obvious than that of sample 3#.

**Figure 7:**
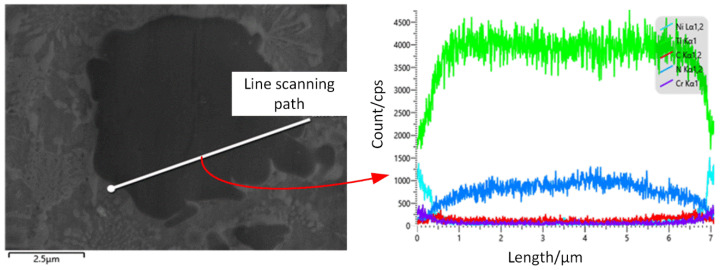
Local line scanning results of sample 4.

To further investigate the substitution reaction of the C element and N element around TiN particles, the line scan of TiN with large particles in sample 4# was carried out individually. As shown in Figure 7, the content of elemental C increases while the content of elemental N decreases around the large particles of TiN. It can be found that the disappearance of elemental N and the appearance of elemental C occur at the edges of the large particles of TiN. A narrow gray band is found around the TiN in the figure, which can be ascribed to the newly formed TiC-enhanced phase, and all these validate the substitution reaction between the N atoms and the C atoms.

### 3.3. Friction Wear Performance Analysis

**Figure 8:**
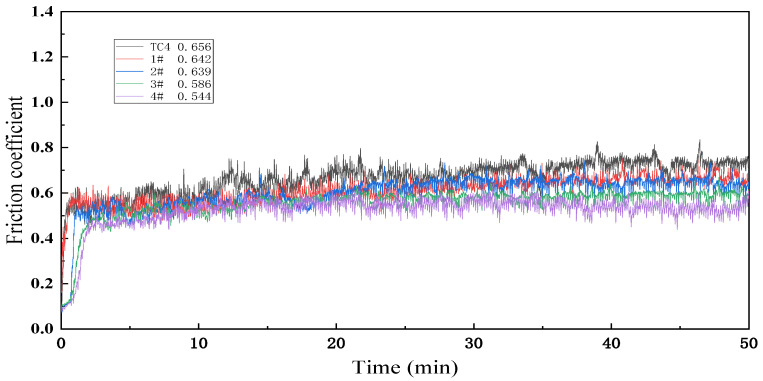
Friction coefficients of the matrix and various samples.

**Figure 9:**
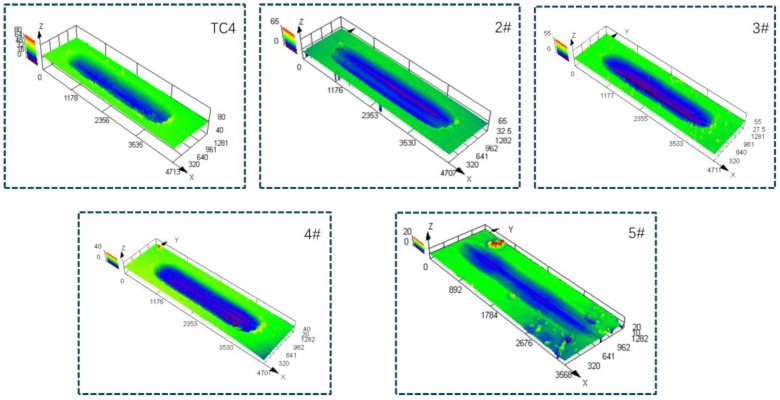
Wear surface morphology of the substrate and various samples.

The friction coefficient and worn surface of each sample are shown in Figure 8 and Figure 9. Tribological properties are one of the important macroscopic characteristics for evaluating the mechanical properties of laser cladding layers. In this study, the wear properties of the laser cladding layers of each sample were tested to evaluate the wear resistance of Ti(C, N)-reinforced composites using the friction coefficient, wear volume, and wear surface morphology as the main performance indexes. To visualize the tribological properties of each sample, the matrix and samples were tested under the same conditions. The friction coefficient curves of different samples 1#–4# are shown in Figure 8. It can be seen that the wear process of each sample went through two wear stages: the pre-stable run-in stage and the stabilization stage (around 20 min). The data of 20 min–50 min are chosen here to calculate the average friction coefficient of each sample. The average values of 0.656, 0.642, 0.639, 0.586, and 0.544 for the substrate and samples #1–4 are in the following order of magnitude: TC4 substrate: > 1# > 2# > 3# > 4#. The coefficient of friction of sample 4# is the smallest, 0.829 times that of the substrate.

**Figure 10:**
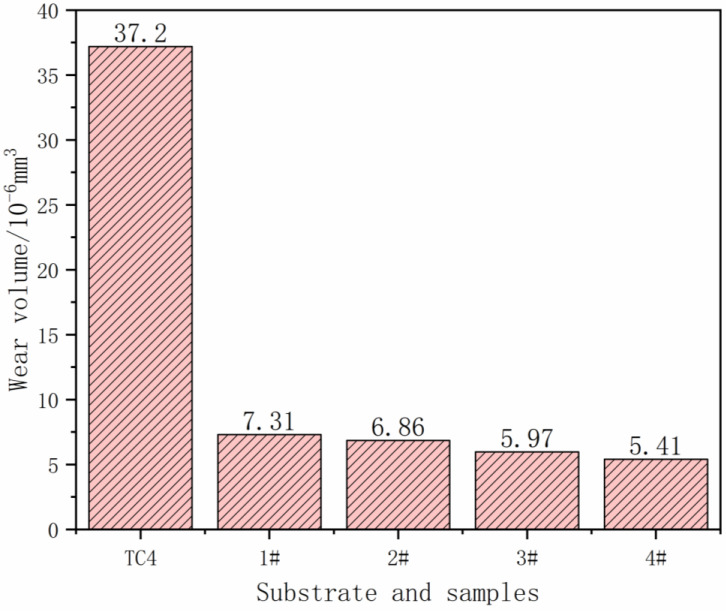
Histograms of wear volume of the substrate and each sample.

The worn surface profiles of each sample are shown in Figure 9. Based on the cross-sectional area of the wear morphology and the wear distance, the wear volume of the matrix and each sample can be calculated, and the results are shown in Figure 10. It can be seen that with the increase in the powder addition ratio, the wear volume of the coating shows a decreasing trend. The wear resistance of the surface coating is improved. The wear volume of sample 4# is the smallest, which is 0.145 times the wear volume of the substrate. The reason is that with the increase in TiN, Ti, and graphite added in the powder, the ceramic phase in the fusion cladding layer, TiC, Ti (C, N), and Ti_2_Ni, and other hard phases play the structural role of the “skeleton”, inhibiting the damage brought about by the micro-cutting to impede the movement of the tearing point of the incision so that the coating has higher abrasion resistance.

### 3.4. Hardness Analysis

**Figure 11:**
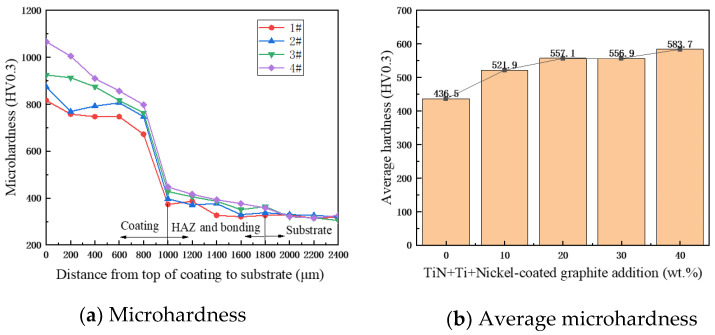
Microhardness and average microhardness on TC4 substrate and specimens 1#–4#.

Microhardness is an important index for evaluating the properties of materials, as shown in Figure 11a, which shows the hardness distribution of specimens 1#–4# from the top of the coating to the matrix TC4. It can be seen that the hardness profile shows a gradual decreasing trend and is divided into three main sections, i.e., the coating zone, the bonding and heat-affected zones, and the matrix zone. The average microhardness of the coatings is shown in Figure 11b. With the increase in TiN + Ti + Ni-encapsulated graphite addition, the average microhardness of the coatings increased from 436.5 to 583.7 HV_0.3_, and the average microhardness of coatings of specimens 1#–4# was 1.62, 1.71, 1.81, and 1.92 times higher than that of the set plate, respectively. The average microhardness of the coatings is proportional to the content of the initial powder TiN + Ti + C. The reasons for this were analyzed to be the in situ synthesis of Ti (C, N) ceramic particles and multiple ceramic phases and the increase in hard phases such as TiNi and CrXCY, which enhanced the coating’s ability to resist deformation. In addition, these hard particles promote grain nucleation and limit the growth of grains, which leads to the refinement of the coating grain size, better strength, and higher microhardness of the coating.

**Figure 12:**
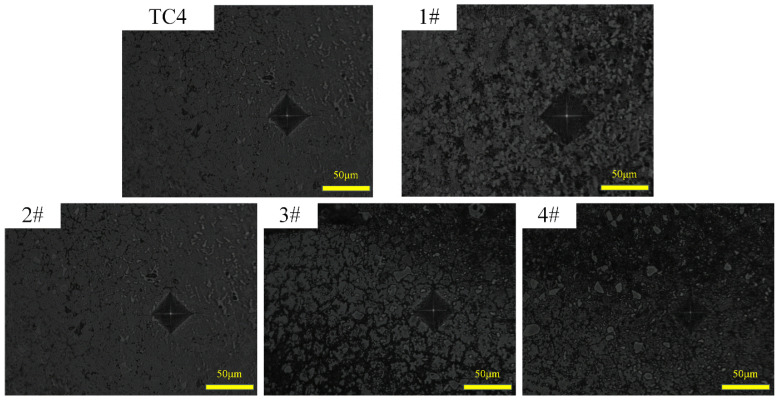
Optical surface morphology of abrasion marks on TC4 substrate and specimens 1#–4#.

Figure 12 shows the typical indentation morphology on the cross-section of the substrate and specimens 1#–4# coatings. It can be noticed that the indentation is regular and there are no microscopic cracks around it, which indicates that the composite coating has a high hardness while still maintaining good toughness.

## 4. Conclusions

In this study, Ti(C, N) ceramic-reinforced nickel-based coatings were prepared by a coaxial powder feed laser cladding. Additionally, the in situ synthesis mechanism and the wear resistance of the coatings were analyzed. The results show the following:

(1) Based on the analysis of thermodynamic calculations, the possible chemical reactions and the priority order during laser cladding were determined. The replacement reaction of TiN and graphite could be carried out when the temperature was higher than 1890 K. Moreover, the diffraction peaks of Ti(C, N) increased with the increase in the addition of TiN and graphite.
(2) The X-ray diffraction (XRD) patterns indicate that the expected ceramic phase was synthesized in situ in the composite coating. Against the thermodynamic analysis, it can be seen that C can displace [N] in TiN at 1900 K, forming Ti(C, N) in the outer layer of large-particle TiN; the crystal structures of TiC and TiN are very similar; and small-particle TiN can form Ti(C, N) solid solution directly. Combined with the previous XRD and thermodynamic analyses, it can be judged to be a Ti(C, N) solid solution formed with C elements after the melting of small-grained TiN.
(3) As TiN, Ti, and graphite powder additions increased, the friction coefficient of the composite coatings decreased and the wear resistance was improved. In addition, the friction coefficient and wear rate of sample 4 are 0.829 and 0.145 times those of the substrate, exhibiting the best wear resistance. The reason is that with the increase in TiN, Ti, and graphite added in the powder, the ceramic phase in the fusion cladding layer, TiC, Ti (C, N) and Ti2Ni, and other hard phases play the structural role of the “skeleton”, inhibiting the damage brought about by the micro-cutting to impede the movement of the tearing point of the incision so that the coating has higher abrasion resistance.
(4) With the increase in TiN + Ti + Ni-encapsulated graphite addition, the average microhardness of the coatings increased from 436.5 to 583.7 HV_0.3_, and the average microhardness of coatings of specimens 1#–4# was 1.62, 1.71, 1.81, and 1.92 times higher than that of the set plate, respectively. The average microhardness of the coatings is proportional to the content of the initial powder, TiN + Ti + C. The reasons for this were analyzed to be the in situ synthesis of Ti (C, N) ceramic particles and multiple ceramic phases and the increase in hard phases, such as TiNi and CrXCY, which enhanced the coating’s ability to resist deformation.

## Figures and Tables

**Table 1:** Powder content and process parameters for laser cladding.

Sample	Component Content (wt.%)				Process Parameters				
	Ni625	TiN	Ti	C	Laser Power	Scanning Speed	Powder Feeding Rate	Track Distance	*Z*-Axis Increment
1#	90	5.08	3.93	0.99	480 W	6.0 mm/s	0.7 r/min	30%	0.32 mm
2#	80	10.16	7.87	1.97					
3#	70	15.24	11.8	2.96					
4#	60	20.32	15.73	3.95					

**Table 2:** Chemical composition of nickel-based alloys and TC4 (wt.%).

Component	Cr	Mo	Nb	Mn	Si	C	Al	V	Ni	Fe	Ti	N
In625	20.0–23.0	8.0–10.0	3.15–4.15	≤0.5	≤0.5	0.02			Bal.	≤5.0		
TC4	-	-	-	-	-	0.10	6.03	4.01	≤0.3	0.3	Bal.	0.01

## Data Availability

The original contributions presented in the study are included in the article, further inquiries can be directed to the corresponding authors.
